# Latest Curriculum of the Apothecaries' Company

**Published:** 1830-10-01

**Authors:** 


### LXVI.

### REGULATIONS TO BE OBSERVED BY STUDENTS, WHOSE ATTENDANCE ON LECTURES SHALL COMMENCE ON OR AFTER THE 1st OF JANUARY, 1831.*

EVERY candidate for a certificate to practise as an apothecary, will be required to produce testimonials of having served an apprenticeship† of not less than five years to an apothecary:

Of having attained the full age‡ of twenty-one years:

Of good moral conduct:§ and,

Of having devoted at least TWO YEARS to an attendance on lectures and hospital practice.

### COURSE OF STUDY.

The Candidate must have attended the following Courses of Lectures:||

| | | |
|---|---|---|
| CHEMISTRY: | Two Courses—Each Course consisting of not less than Forty-five Lectures. | |
| MATERIA MEDICA, & THERAPEUTICS: | Two Courses—Each Course consisting of not less than Forty-five Lectures. | |
| ANATOMY and PHYSIOLOGY: | Two Courses: | Of the same extent as required by the Royal College of Surgeons, London. |
| ANATOMICAL DEMONSTRATIONS: | Two Courses: | |

* Students who are at present pursuing their medical studies, and those who may begin to attend lectures at the commencement of the next medical session, (viz. October,) will be received as candidates for examination by complying with the regulations heretofore published.

† The apprenticeship must have been served with a person legally qualified to practise as an Apothecary, either by having been in practice prior to or on the 1st of August, 1815, or by having received a certificate of his qualification from the Court of Examiners.

‡ As evidence of age, a copy of the baptismal register will be required in every case where it can possibly be procured.

§ A testimonial of moral character from the gentleman to whom the candidate has been an apprentice, will always be more satisfactory than from any other person.

|| The lectures required in each course respectively, must be given on separate days.

| | | | |
|---|---|---|---|
| PRINCIPLES and PRACTICE of MEDICINE: | Two Courses—Each Course consisting of not less than Forty-five Lectures, to be attended subsequently to the termination of the first Course of Lectures on Chemistry, Materia Medica, and Anatomy and Physiology. | | |
| BOTANY: | One Course. | | |
| MIDWIFERY: and The DISEASES of WOMEN and CHILDREN: | Two Courses: | To be attended during the second year. | |
| FORENSIC MEDICINE: | One Course: | | |

Students are moreover recommended diligently to avail themselves of instruction in *morbid anatomy.*

The candidate must also have attended for *twelve months,* at least, the physician's practice at an hospital containing not less than sixty beds, and where a course of clinical lectures is given; or for *fifteen months* at an hospital wherein clinical lectures are not given; or for *fifteen months* at a dispensary connected with some medical school recognized by the Court. The whole of such attendance to be *subsequent* to the first year of attendance on lectures.

---

